# Influence of the Extraction Method on the Biological Potential of *Solidago virgaurea* L. Essential Oil and Hydrolates

**DOI:** 10.3390/plants13162187

**Published:** 2024-08-07

**Authors:** Marko Malićanin, Ivana Karabegović, Natalija Đorđević, Stojan Mančić, Sandra Stamenković Stojanović, Duško Brković, Bojana Danilović

**Affiliations:** 1 Faculty of Agriculture, University of Niš, Kosančićeva 4, 37000 Kruševac, Serbia; malicanin.marko@ni.ac.rs; 2 Faculty of Technology, University of Niš, Bulevar oslobodjenja 124, 16000 Leskovac, Serbia; ivana.karabegovic@tf.ni.ac.rs (I.K.); natalija@tf.ni.ac.rs (N.Đ.); stojan@tf.ni.ac.rs (S.M.); sandrastamenkovic@tf.ni.ac.rs (S.S.S.); 3 Faculty of Agronomy in Čačak, University of Kragujevac, Cara Dušana 34, 32000 Čačak, Serbia; dbrkovic@kg.ac.rs

**Keywords:** *Solidago virgaurea* L., microwave-assisted hydrodistillation, solvent-free extraction, biological activity

## Abstract

*Solidago virgaurea* L., or European goldenrod, has a long tradition in folk medicine due to the wide range of its biological activity. This paper aimed to investigate the antimicrobial and antioxidative potential of *S. virgaurea* essential oil and hydrolates obtained by traditional and novel extraction techniques. For that purpose, hydrodistillation, microwave-assisted hydrodistillation and solvent-free extraction were performed. Analysis of the composition of essential oils indicated the presence of 59 different compounds with cyclocolorenone, germacrene D and spathulenol being the dominant in all essential oil samples. Antimicrobial activity was detected in all the analyzed samples, with higher effect on Gram-positive microorganisms compared to Gram-negative. Regarding the type of performed extraction process, the introduction of microwaves induced higher antimicrobial and antioxidative potential in both essential oils and hydrolates. Hydrolates obtained in microwave-assisted processes had pronounced antioxidative activity, which creates a good basis for further investigation of this side product’s potential use in the food, cosmetic and pharmaceutical industries.

## 1. Introduction

Essential oils (EOs) represent natural, aromatic and volatile secondary plant metabolites, insoluble in water. They are usually liquid at room temperature, colorless and have a characteristic smell [1]. The various uses of EOs from different plants have been known since the Middle Ages. Their antimicrobial, antioxidant, anti-inflammatory, anti-parasitic, anti-tumor and other effects have been proven through practice and numerous scientific research studies [2]. In recent times, EOs have gained importance in the various branches of industry. In the food industry, the development of resistance of microorganisms to the action of synthetic preservatives threatens to become a global problem today. In order to obtain safe foods of acceptable quality, it would be necessary to use increased amounts of synthetic preservatives [3]. Research has shown that overcoming the potential health danger caused by the use of high doses of synthetic preservatives can be achieved with the use of EOs in food systems [4,5].

EOs have a complex chemical composition, which can contain hundreds of compounds mostly from the group of terpenes, terpenoids, phenolics and other low-molecular-weight compounds. The diversity in the chemical composition is related to the EOs’ pronounced biological activity [6]. The most commonly applied extracting method of EOs from dried plant material is hydrodistillation (HD). Since it uses water as a solvent, HD is considered an environmentally friendly method, but it requires a significant amount of energy. Therefore, including microwaves in the extraction process can significantly shorten the extraction time and improve the yield of EOs [7]. On the other hand, solvent-free extraction enables obtaining EOs from the raw plant material without the addition of water or other solvents, which avoids the problem of disposal of waste water [8]. The EO extracting method can significantly affect the chemical composition and thus the biological potential [9,10,11]. 

Hydrolates or hydrosols remain as side products of the EO extraction process from various plant materials [12]. Hydrolates contain residual EO that is usually discarded. However, a lot of aromatic compounds are retained in hydrolates, emphasizing their potential for use in various food and pharmaceutical products [13]. The chemical composition of the hydrolate is usually similar to that of the EO of the same plant. However, the quantitative composition may differ. Often, the main components of EO and hydrolates can be different. Also, some compounds that are not present in EOs can be identified in hydrolates [14]. Since hydrolates contain an abundance of aldehydes, ketones, alcohols and phenolic compounds, they have shown good biological activity with the potential for further application for food, cosmetic and pharmaceutical purposes [15]. 

The genus *Solidago* belongs to the *Asteraceae* family and includes over 100 species distributed in Europe, Asia, and North and South America. The common name for the species of *Solidago* L. genius is “goldenrods”, and it represents perennial herbaceous plants that can be found in mountainous, plain and rocky areas [16]. The most studied species in Europe is *Solidago virgaurea* L., according to the European Medicines Agency [17]. *S. virgaurea* L. is a naturally growing ramified plant with a round stem and yellow florets, reaching a height of up to 1 m. *S. virgaurea* L. essential oil and extracts are extremely rich in a variety of bioactive compounds, which explains their application in traditional medicine as an anti-inflammatory agent, preventing infections and improving the immune system and general health [18].

Although the biological activity of *S. virgaurea* has been extensively investigated [19,20,21,22,23,24], to the authors’ knowledge there are scarce data on the analysis of *S. virgaurea* essential oil and hydrolates obtained by novel extraction techniques. This work aimed to analyze the influence of the different extraction method on the chemical composition and biological activity of *S. virgaurea* L. essential oils and the potential of the use of such obtained hydrolates. For that purpose, three different extraction methods have been applied: hydrodistillation, microwave-assisted hydrodistillation and solvent-free extraction.

## 2. Results and Discussion

### 2.1. GC/MS Analysis of EOs

GC/MS analysis of *S. virgaurea* essential oil indicated the presence of five identified groups of compounds (Table 1). A total number of 59 components were identified in the samples of *S. virgaurea* essential oil obtained by hydrodistillation (EGEO1), microwave-assisted hydrodistillation (EGEO2) and solvent-free microwave-assisted extraction (EGEO3), with a recovery of 91.2–93.3%. The application of microwaves during the extraction increased the number of detected components, so 52 components were identified in the EGEO2 and 50 in the EGEO3 sample. On the other hand, the use of conventional hydrodistillation resulted in 45 identified compounds in GEO1. The most abundant compound in all analyzed samples was cyclocolorenone, with 29.5, 18.0 and 23.6% in EGEO1, EGEO2, and EGEO3, respectively. Cyclocolorenone belongs to the group of aromadendrene-type sesquiterpenes, and it is known for good phytotoxic, antibacterial and antifungal activity against *B. subtilis*, *B. cereus*, *M. thermosphactum*, *E. coli*, *E. cloacae*, *C. freundii*, *C. lunata*, and *C. cochliodesspinusum* [25]. The presence of cyclocolorenone can also be connected with anti-inflammatory activity [26], probably due to the α,β unsaturated cyclopentenone ring, which can contribute to the interaction with cysteine and glutathione and evince a pharmacological effect [27]. This compound was isolated from *Solidago canadensis* [27], *Magnolia grandiflora* [25] and *Critonia aromatisans* [26]. On the other hand, some researchers have not reported the presence of this compound in *S. virgaurea* essential oil [24,28].

Cyclocolorenone, together with Germacrene D, were found to be the most abundant constituents in *S. canedensis* essential oil [27]. Germacrene D was detected in all three essential oils in the amount of 7.7–9.4%, which is similar to 6–8% of germacrene present in essential oil isolated from *S. virgaurea* flowering tops from the Russian Altai [22]. Additionally, previous studies of extracts and hydrodistillates of wild-grown *S. canadensis* indicated this compound as their main constituent [30]. Germacrene D is a monocyclic sesquiterpene usually present in different plant essential oils. It can be a precursor to many other sesquiterpenes, and possesses a wide range of biological activity including substantial antimicrobial activity [31,32]. 

Spathulenol belongs to the group of oxygenated sesquiterpenes and was present in the amount of 4.8–5.9% in the analyzed samples. This compound has great antimicrobial and immunomodulatory potential, as reported by Ziaei et al. [33] and Cazella et al. [34]. The mode of action can be explained by the high hydrophobicity of sesquiterpenes, which allows easier penetration through the cell membrane and further interaction with proteins and organelles in intracellular space [35].

It is interesting to note that microwave application during EO isolation induced the increase in the presence of spathulenol, Germacrene D and Germacra-4(15),5,10(14)-trien-1-α-ol, while it decreased the detected concentration of cyclocorenone. It has already been reported that it appears that the extraction method significantly influences the chemical composition of essential oil and can lead to the dominance of different components [10,11,27]. Extraction techniques which involved microwaves application induced a higher content of oxygen-containing sesquiterpenes in EGEO 2 and EGEO3 compared to hydrodistillation. This may occur due to the short time of heating, which could lead to an excess of oxygenated compounds [36].

### 2.2. UHPLC-DAD-ESI/MS Analysis of Hydrolates

The list of phenolic compounds detected in the *S. virgaurea* hydrolates obtained in the hydrodistillation (EGH1), microwave-assisted hydrodistillation (EGH2) and solvent-free microwave-assisted process (EGH3) is shown in Table 2. Among the 38 numerated compounds, two main classes were detected and identified in the samples of the extracts, phenolic and other acids and their glycosides (comp. no. 1, 3–20) and flavonoids (comp. no. 21–33). Some of the compounds were tentatively identified according to both their UV-Vis and MS/MS spectra (comp. no. 4, 5, 12, 25 and 28). Five compounds were not identified (34–38), and partially identified as phenolic acids derivatives were comp. no. 17 and 20. There were major contributions from the quercetin rhamnoside derivative and quercetin rhamnoside in all three hydrolates (comp. 28 and 29, respectively) and caffeic acid and quercetin arabinoside (or xyloside, comp. no. 16 and 26, respectively) were also dominant in the EGH2 and EGH3. One hydroxy-coumarin, assigned as methylumbelliferyl glucuronide was also detected in the EGH1 (comp. no. 2).

Hydrolates of *S. virgaurea* plants are rich in compounds with pronounced biological activity. Some pharmacological studies showed good antimicrobial and antioxidant activity of quinic acid [45], as well as quercetin and its derivates [46], whose presence was detected in all three hydrolates. Citric acid, also present in all of them, is a compound with proven anti-oxidative and antimicrobial activity, with an important role in the body as an anti-inflammatory agent [47]. Other studies reported the content of quercetin and kaempferol and their derivatives in extracts of *S. virgaurea* from Poland, Italy, Hungary, and Romania [18]. In addition to flavonoids, the presence of caffeic acid and chlorogenic acid and their derivatives was also recorded, which is in agreement with the obtained results.

### 2.3. Antimicrobial Activity

The results of the evaluation of antimicrobial potential indicated that Gram-positive microorganisms were generally more susceptible to the action of EGEOs and EGHs (Table 3 and Table 4). Similar results were observed for methanolic extract of *S. vigaurea* from Turkey [19]. Among Gram-negative strains, *E. coli* was the least resistant to the analyzed samples of EOs and hydrolates. As expected, essential oils had a much higher antimicrobial effect compared to hydrolates. In most cases, the isolation method did not have significant influence on the antimicrobial activity of EOs against Gram-negative strains. No significant difference was observed for *B.cereus*, *P. vulgaris*, *P. aeruginosa* and *K. pneumoniae*, regardless of the applied EO isolation method.

On the other hand, the antimicrobial activity of hydrolates was influenced by the performed production method for all other strains except *B. cereus* and *C. albicans*. EO obtained by microwave-assisted hydrodistillation, as well as its matching hydrolate, had the best antimicrobial activity against some strains, indicating that the combination of hydrodistillation and microwaves increases the antimicrobial potential. This could be connected to the highest number of isolated compounds in the EGEO2 and the highest content of oxygenated sesquiterpenes, which are potent antimicrobial agents [35]. In the examination of EOs of *S. virgaurea* collected at the altitudes of 290 m and 650 m, poorer antimicrobial activity was observed, indicating good activity against *S. aureus*, but low activity against *E. coli*, and no effect on *P. aeruginosa* [22]. The antimicrobial activity of hydrolates reported in this research can be considered substantial, taking into consideration that water extracts of *S. virgaurea* from France showed no activity against *E. coli*, *S. aureus*, *P. aeruginosa*, *S. mutans*, *S. salivarius*, *E. faecalis* and *C. albicans* [23].

### 2.4. Antioxidant Activity of Solidago virgaurea L. Essential Oils and Hydrolates

The results of the antioxidant activity of *S. virgaurea* L. EOs obtained by three extraction methods are shown in Table 5. According to the obtained results, EGEO3 has the most pronounced antioxidant activity. The neutralization of DPPH radicals after 20 min of incubation was 76.11%, which is statistically significantly different, compared to EOs obtained by other extraction methods (*p* < 0.05).

A significant difference in antioxidant activity was also recorded with the use of EOs obtained by hydrodistillation and microwave-assisted hydrodistillation. Namely, the neutralization of DPPH radicals of these essential oils was 58.48% and 62.23% for EGEO1 and EGEO2, respectively. Other results also showed good antioxidant activity of the species from the same genus, i.e., *S. canadensis* EO at a concentration of 7.82 mg/mL neutralized 50% of DPPH radicals in 30 min [48].

The antioxidant activity of EOs originates from the presence of hydrocarbon and oxygenated monoterpenes and sesquiterpenes [49]. The largest percentage in the chemical composition in EGEO1, EGEO2 and EGEO3 were hydrocarbon and oxygenated sesquiterpenes, with content in the range of 14.7–15.3% and 25.5–32.2%, respectively (Table 1). These groups include compounds with proven antioxidant activity also detected in other EOs: germacrene D [50], γ-muurolene [51], and spathulenol [52]. Although monoterpenes had a significantly smaller share in the chemical composition, with concentrations below 1%, their contribution to the antioxidative activity should not be neglected. However, small amounts of such compounds can act synergistically and significantly affect and improve the overall antioxidant potential [53].

A significant antioxidant potential of the obtained hydrolates was recorded (Table 6). The low concentration (1 mg/mL) of EGH3 neutralized DPPH radicals by 50.32% after 20 min of incubation. EGH3 showed a significant difference in antioxidant activity compared to other hydrolates. The same concentration of EGH2 neutralized 45.75% of radicals during the same time of incubation. Finally, EGH1 had the least-pronounced effect, with an inhibition of DPPH radicals of 35.71%.

The literature data show a good antioxidant activity of the aqueous extract of *S. virgaurea* L. from Turkey, which neutralized 30.67% of DPPH radicals at the concentration of 100 μg/mL, while the methanol extract shows almost twice the effect (64.26%) [19]. A good antioxidant potential of the aqueous extract of *Solidago graminifolia* originating from Romania was also recorded [54]. On the other hand, no statistically significant difference was recorded in the inhibition of DPPH radicals by aqueous extracts of *S. virgaurea* from the Czech Republic obtained by pressurized fluid extraction and ultrasonic extraction [55]. 

In this study, hydrolates showed a higher antioxidative potential compared to EOs. Other studies have also shown that hydrolates have the capability to inhibit DPPH radicals in a lower concentration than EOs [14]. The pronounced antioxidant activity of the hydrolates can be related to the nonvolatile TPC, which are antioxidant agents. Goldenrods are considered a rich source of phenols (Table 2) which have the ability to regenerate their chemical structure, and thus their antioxidant potential [19]. The presence of compounds such as citric, gallic, and chlorogenic acids most likely significantly contributed to the increased antioxidant power of the hydrolates. Results for TPC show that the order of increasing antioxidant activity for hydrolates is proportional to TPC, expressed in mg GAE/g (Table 5). The largest TPC was in EGH3, and amounted to 7.83 mg GAE/g. Other studies have shown a high content of phenol in the aqueous extract of *S. graminifolia* [53], as well as in aqueous extracts of *S. virgaurea* obtained by supercritical fluid extraction and pressurized liquid extraction (98.0–142.0 mg GAE/g) [20]. The qualitative and quantitative content of phenolic compounds in goldenrods can vary, depending on the growing conditions of the plant, the part of the plant used for extraction, and the processing and extraction conditions [21].

## 3. Material and Methods

### 3.1. Plant Material

Fresh plant material (*Solidago virgaurea* L.) was collected at the Suvobor, Koštunići (866 m a.s.l, 44°7′17″ N 20°10′55″ E ), near Čačak, Serbia and identified by Duško Brković, a botanist from the University of Kragujevac. Plant specimens are deposited in the Herbarium of the Botanical Garden “Jevremovac” in Belgrade, part of the University of Belgrade, under the number 4093 BEOU. Harvest was performed in the flowering period during August and September and part of the plant material was air-dried for 20 days at room temperature.

### 3.2. Essential Oil and Hydrolate Isolation 

*S. virgaurea* EO was isolated from the flowering part of the plant by three different methods. Conventional isolation of European goldenrod EO was performed by hydrodistillation method on Clavenger apparatus. An amount of 50 g of plant material was mixed with 500 mL of water in a 1 L glass flask and heated for 120 min at boiling temperature in a 1l heating mantle (HEAM-1K1-001, Labbox Labware S.L., Barcelona, Spain). Microwave-assisted hydrodistillation of European goldenrod EO was conducted in a 1 L glass flask by heating the 50 g of plant material and 500 mL of water in the presence of microwaves (800 W) in a microwave oven (Beko, MWC 2000 MW) connected to the Clavenger apparatus. The isolation was performed for 60 min, continuously. For solvent-free microwave-assisted extraction of EO, plant material was immersed in distilled water for 15 min and then subjected to microwave-assisted extraction continuously, for 60 min, in a 1 L glass flask. All isolation procedures were performed in triplicate. EOs were collected from the Clavenger apparatus tube in a 5 mL glass flask and dehydrated anhydrous sodium sulfate. After the separation of essential oils, hydrolate samples were collected and labelled as EGH1—hydrolate from conventional hydrodistillation, EGH2—hydrolate from microwave-assisted hydrodistillation, and EGH3—hydrolate obtained by solvent-free microwave-assisted extraction. Hydrolates and EOs were stored at 4 °C until further analysis.

### 3.3. Determination of EO Composition

Determination of EO composition was performed by gas chromatography/mass spectroscopy (GC/MS) and gas chromatography/flame ionization detection (GC/FID) analysis under identical conditions, as already described [10] on the Agilent Technologies 7890B gas chromatograph, equipped with nonpolar, silica capillary column, HP-5MS (5% diphenyl- and 95% dimethyl-polysiloxane, 30 m × 0.25 mm, 0.25 μm film thickness; Agilent Technologies, Santa Clara, CA, USA) coupled with inert, selective 5977A mass detector of the same company. MSD ChemStation, MassHunter Qualitative Analysis and AMDIS_32 software (Agilent Technologies, Santa Clara, CA, USA) was used for data analysis. Quantification of the components was performed by the use of external standards in the concentration ranges as follows: 1,8-cineole (0.25–3 mg/mL), limonene (0.5–4 mg/mL), linalool (1.67–15 mg/mL), thymol (1.78–16 mg/mL) and γ-terpinene (0.75–5 mg/mL). 

### 3.4. UHPLC-DAD-ESI/MS Analysis of Hydrolates

The hydrolates were analyzed qualitatively using liquid chromatography with ultra-high performance (UHPLC), following Zvezdanović’s [40] method. The Dionex Ultimate 3000 UHPLC+ system, with a DAD detector and LCQ Fleet Ion Trap Mass Spectrometer (Thermo Fisher Scientific, Waltham, MA, USA) and a Hypersil gold C18 column (50 × 2.1 mm, 1.9 μm), was utilized for analysis. The column was tempered at 25 °C with a mobile phase made up of two solvents, 0.1% formic acid in water (A) and methanol (B), flowing at a rate of 0.250 mL/min. DAD signal estimate was performed using three detection wavelengths: 300, 330, and 350 nm. A 3D ion trap with electrospray ionization (ESI) in negative ionization mode was used for mass spectrometry analysis. Full-range MS spectra were obtained at *m*/*z* 100–900, followed by a data-dependent scan to analyze the collision-induced dissociation of identified molecular ion peaks ([M − H]^−^) adjusted at 30 eV. Xcalibur software (version 2.1) was used to control the instrument, acquire data, and analyze them. Data were further processed using Origin 7.5 software. The detected compounds were identified using their retention periods, UV–Vis spectra from the DAD detector, and MS spectra with the matching molecular ion peaks, as well as the distinctive ion fragmentation of chosen peaks (MS/MS) from the corresponding UHPLC chromatograms. The discovered chemicals were identified using reference standards for some of them. The resulting data were compared to those available in the literature.

### 3.5. Determination of Total Phenolic Content

To determine the total phenolic content (TPC), the volume of 0.5 mL of each hydrolate was added to 4.5 mL of distilled water and 0.5 mL of Folin–Ciocalteu reagent and left for 5 min at room temperature in a dark place. Afterwards, 5 mL of 7.5% sodium carbonate was added and left in a dark place for 90 min at room temperature. Absorbance was recorded at 765 nm, and a mixture of 5 mL of distilled water, 0.5 mL Folin–Ciocalteu reagent and 5 mL 7.5% sodium carbonate was used as blank. In order to create a calibration curve, the whole procedure was repeated with concentrations of gallic acid in the range of 30 µg/mL to 300 µg/mL, and the results were expressed in mg equivalent of gallic acid per g of dry plant material [56].

### 3.6. Determination of Antioxidant Activity 

The DPPH assay was used for the determination of the antioxidant activity of the EOs and hydrolates [11]. *S. virgaurea* EOs were dissolved in methanol at a concentration of 25 mg/mL, and hydrolates at a concentration of 1 mg/mL. Methanol solution of 2,2-diphenyl-1-picrylhydrazyl (DPPH) (Sigma-Aldrich, Sternheim, Germany) radical (1 mL, 3 × 10^−4^ mol/L) was added to 2.5 mL of EO and hydrolate solution. The absorbance of the samples was determined at 517 nm (A_S_) at a spectrophotometer (2100 UV spectrophotometer, Cole-Parmer, IL, USA), after 20 min of incubation in a dark place. Absorbance was also determined for the control sample, which was a mixture of 1 mL DPPH and 2.5 mL methanol (A_C_) and for the untreated EO and hydrolate samples (A_B_). Methanol was used as a blank. The degree of reducing of free radicals was calculated according to the equation

DPPH radical reducing ability (%) = 100 − [(A_S_ − A_B_) × 100/A_C_] (1)

The experiment was performed in triplicate for each sample individually, and the results were presented as the average value ± standard deviation.

### 3.7. Antimicrobial Activity

For evaluation of the antimicrobial activity of the EOs and hydrolates, the determination of minimal inhibitory concentration (MIC) was performed according to Clinical and Laboratory Standard Institute (2012) protocols [57] against 4 Gram-positive strains (*Bacillus subtilis* ATCC 6633, *Bacillus cereus* ATCC 11778, *Listeria monocytogenes* ATCC 15313 and *Staphylococcus aureus* ATCC 25923), 4 Gram-negative strains (*Escherichia coli* ATCC 25922, *Proteus vulgaris* ATCC 8427, *Pseudomonas aeruginosa* ATCC 27853 and *Klebsiella pneumoniae* ATCC 700603) and one yeast (*Candida albicans* ATCC 2091). Appropriate dilutions of extracts and EOs were transferred to microtiter plates with 50 µL of medium, Mueller–Hinton Broth (“Torlak”, Belgrade, Serbia) for bacteria and Sabouraud maltose Broth (“Torlak”, Belgrade, Serbia). A volume of 50 µL of microorganism suspension in sterile saline containing 1 × 10^6^ CFU/mL was transferred into the wells of microtiter plates. Microtiter plates were incubated at 37 °C for 24 h for bacteria and 72 h at 25 °C for fungi, and the results were recorded by EZ read 400 Elisa microplate reader (Biochrom, Meckenheim, Germany). 

### 3.8. Statistical Analysis

All the experiments were carried out in triplicate and presented as the average value ± standard deviation. Statistically significant difference among the results was calculated by software SPSS 21.0 (IBM, Armonk, NY, USA), using one-way ANOVA followed by Tukey’s multiple comparison test. Results were considered significantly different if the *p*-value was lower than 0.05.

## 4. Conclusions

The obtained results indicated great potential for the possible use of *S. virgaurea* essential oil and hydrolates. The application of novel extraction processes significantly contributes to the increase in antimicrobial and, especially, antioxidative capacity of *S. virgaurea* essential oil and hydrolates. The microwave-assisted extraction process gives multiple advantages and produces essential oil and hydrolates with more pronounced biological activity compared to the hydrodistillation. According to their good antimicrobial and antioxidative potential, analyzed essential oils and hydrolates can be used in the production of biodegradable packaging in the food industry or as additives in the cosmetic industry for increased antioxidative potential of the cosmetic products. It is important to emphasize the possibility of the use of hydrolates, which are usually side products in essential oil extraction. Further research is essential in the development of products with incorporated *S. virgaurea* essential oil and hydrolates.

## Figures and Tables

**Table 1:** Results of GC/MS analysis of *S. virgaurea* essential oil obtained by hydrodistillation (EGEO1), microwave-assisted hydrodistillation (EGEO2) and solvent-free microwave-assisted extraction (EGEO3).

No.	t_ret_, min	Compound	Molecular Formula	RI^exp^	RI^lit^	Concentration, %		
						EGEO1	EGEO2	EGEO3
1	6.63	*α*-Pinene	C_10_H_16_	930	932	0.6	0.2	0.1
2	7.07	Camphene	C_10_H_16_	946	946	0.2	tr	tr
3	7.95	*β*-Pinene	C_10_H_16_	975	974	0.1	tr	-
4	9.55	*ρ*-Cymene	C_10_H_14_	1025	1020	0.1	0.1	tr
5	9.67	Limonene	C_10_H_16_	1028	1024	-	tr	-
6	12.53	Linalool	C_10_H_18_O	1104	1095	0.8	1.0	1.0
7	13.49	*α*-Campholenal	C_10_H_16_O	1127	1122	-	tr	-
8	14.23	Camphor	C_10_H_16_O	1145	1141	0.2	0.3	0.2
9	14.4	2-(1*Z*)-propenyl-phenol	C_9_H_10_O	1149	1146	0.3	0.2	0.2
10	14.64	Menthone	C_10_H_18_O	1155	1148	0.5	0.6	0.6
11	15.07	*iso*-Menthone	C_10_H_18_O	1165	1158	-	0.2	0.2
12	15.27	*neo*-Menthol	C_10_H_20_O	1170	1161	-	-	0.1
13	15.36	Borneol	C_10_H_18_O	1172	1165	0.5	0.6	0.4
14	15.62	Menthol	C_10_H_20_O	1179	1167	1.1	1.4	1.5
15	16.4	Dihydro carveol	C_10_H_18_O	1197	1192	0.2	0.2	-
16	16.6	*ρ*-Cymen-9-ol	C_10_H_14_O	1202	1204	-	0.2	0.1
17	18.27	Pulegone	C_10_H_16_O	1241	1233	-	0.1	0.1
18	18.51	Carvone	C_10_H_14_O	1246	1239	-	0.3	tr
19	18.82	*trans*-Sabinene hydrate acetate (Ac vs. IPP)	C_12_H_20_O_2_	1254	1253	-	-	0.1
20	20.12	Bornyl acetate	C_12_H_20_O_2_	1284	1283	3.1	5.2	3.6
21	20.37	(*E*)-Anethole	C_10_H_12_O	1290	1282	-	0.1	-
22	20.5	Menthyl acetate	C_12_H_22_O_2_	1293	1294	-	-	tr
23	21.66	*ρ*-vinyl-Guaiacol	C_9_H_10_O_2_	1320	1309	0.9	0.9	1.1
24	24.59	*β*-Elemene	C_15_H_24_	1390	1389	0.3	0.2	0.2
25	25.24	*β*-Longipinene	C_15_H_24_	1407	1400	1.6	1.5	1.6
26	25.71	(*Z*)-Caryophyllene	C_15_H_24_	1418	1408	0.5	0.5	0.5
27	26.08	*β*-Copaene	C_15_H_24_	1427	1430	0.2	0.2	0.2
28	27.81	*γ*-Gurjunene	C_15_H_24_	1470	1475	1.3	1.4	1.5
29	28.03	*γ*-Muurolene	C_15_H_24_	1476	1478	0.9	0.9	0.8
30	28.22	Germacrene D	C_15_H_24_	1480	1480	7.7	8.8	9.4
31	28.46	*γ*-Himachalene	C_15_H_24_	1486	1481	0.7	0.6	0.5
32	28.8	*α*-Selinene	C_15_H_24_	1495	1498	-	-	0.5
33	29.52	*γ*-Cadinene	C_15_H_24_	1513	1513	0.6	0.6	0.4
34	29.84	*δ*-Cadinene	C_15_H_24_	1522	1522	0.9	1.0	0.7
35	30	Myristicin	C_11_H_12_O_3_	1526	1517	0.8	0.9	0.7
36	30.69	*α*-Calacorene	C_15_H_20_	1544	1544	-	0.2	-
37	31.08	(*E*)-Veltonal	C_14_H_22_O	1554	1555	-	0.2	-
38	31.24	*β*-Vetivenene	C_15_H_22_	1558	1554	1.6	2.2	1.9
39	31.56	Germacrene D-4-ol	C_15_H_26_O	1566	1574	1.2	1.4	1.1
40	31.7	Palustrol	C_15_H_26_O	1570	1567	1.0	1.3	1.3
41	32.17	Spathulenol	C_15_H_24_O	1582	1577	4.8	5.6	5.9
42	32.39	*β*-Copaen-4-*α*-ol	C_15_H_24_O	1588	1590	2.2	3.0	2.5
43	32.64	Salvial-4(14)-en-1-one	C_15_H_24_O	1594	1594	2.2	3.3	2.8
44	33.05	Khusimone	C_14_H_20_O	1605	1604	2.2	2.7	3.1
45	33.2	Hillone	C_15_H_20_O_3_	1609	1607	1.4	2.0	2.0
46	33.44	β-Atlantol	C_15_H_24_O	1616	1608	3.2	4.2	3.8
47	33.77	*β*-Cedrene epoxide	C_15_H_24_O	1625	1621	-	0.7	0.7
48	34.1	Selina-3,11- dien-6-*α*-ol	C_15_H_24_O	1634	1642	0.9	1.5	1.2
49	34.54	Cubenol	C_15_H_26_O	1646	1645	0.5	0.7	0.6
50	34.7	3-Thujopsanone	C_15_H_24_O	1650	1653	1.3	1.5	1.4
51	34.88	Agarospirol	C_15_H_26_O	1655	1646	1.6	1.7	1.7
52	35.01	Gymnomitrol	C_15_H_24_O	1659	1658	4.3	5.0	4.8
53	35.97	Khusinol	C_15_H_24_O	1684	1679	0.4	0.6	0.7
54	36.23	Germacra-4(15),5,10(14)-trien-1-*α*-ol	C_15_H_24_O	1692	1685	5.8	6.8	6.7
55	37.21	Tasmanone	C_14_H_20_O_4_	1719	1726	0.2	-	-
56	38.67	Cyclocolorenone	C_15_H_22_O	1761	1759	29.5	18.0	23.6
57	38.94	14-oxy-*α*-Muurolene	C_15_H_22_O	1769	1767	1.4	-	-
58	39.88	2-*α*-acetoxy-Amorpha-4,7(11)-diene	C_15_H_24_O	1796	1805	0.4	-	0.7
59	41.38	o-methyl, *α*-Pipitzol	C_16_H_22_O_3_	1841	1840	1.0	0.6	0.5
Total identified (%)						91.2	91.4	93.3
Grouped components (%)								
Monoterpene hydrocarbons (1–3, 5)						0.9	0.3	0.1
Oxygen-containing monoterpenes (7–8, 17)						0.2	0.4	0.3
Sesquiterpene hydrocarbons (24–34)						14.7	15.7	16.3
Oxygen-containing sesquiterpenes (41–43, 46–48, 50, 52–54, 58)						25.5	32.2	31.2
Aromatic compounds (4, 6, 9–16, 18–23, 36–40, 44–45, 49, 51, 55–59)						48.6	42	44.7
Others (35)						0.8	0.9	0.7

t*_ret_*: Retention time; RI^lit^—Retention indices from the literature [29]; RI^exp^—Experimentally-determined retention indices using a homologous series of *n*-alkanes (C8-C20).

**Table 2:** Results of UHPLC-DAD-ESI/MS analysis of *S. virgaurea* hydrolates obtained in the hydrodistillation (EGH1), microwave-assisted hydrodistillation (EGH2) and solvent-free microwave-assisted process (EGH3).

No.	*t*_R_, min	UV/Vis Data from UHPLC-DAD Signal Absorb. max., nm	Molecular Ion [M − H]^–^ *m*/*z*	MS/MS Fragment Ions	Assignment [Reference]	Sample		
						EGH1	EGH2	EGH3
1	0.79	-	191	173, 11 (100%)	Quinic acid [37]	+	+	+
2	0.86	-	351	333, 291, 193, 185, 175 (100%)	Methylumbelliferyl glucuronide (MB: SM832751 *)	-	+	-
3	0.97	-	191	173, 111 (100%), 85	Citric acid (standard)	+	+	+
4	1.10	-	371	353, 191 (100%), 185, 179, 173, 135	Hydroxy derivative of caffeoyl quinic acid, isomer, *tent*.	+	-	+
5	1.24	-	371	353, 191 (100%), 179, 173, 135	Hydroxy derivative of caffeoyl quinic acid, isomer, *tent*.	+	-	+
6	1.36	-	169	151 (100%)	Gallic acid (standard, [38])	+	-	+
7	1.67	-	169	151 (100%)	Trihydroxybenzoic acid (PubChem CID: 11974 **)	+	-	+
8	1.80	259 298 322	315	153 (100%)	Protocatechuic acid hexoside [39]	+	+	-
9	2.00	-	353	191 (100%), 179, 173, 135	Neochlorogenic acid, cis isomer (standard)	+	-	+
10	2.21	217 261 295	153	-	Protocatechuic acid [40]	+	+	-
11	2.50	327 298	353	191 (100%), 179, 173, 161, 135	Neochlorogenic acid, trans isomer (standard)	+	-	+
12	3.10	251	447	429, 315, 271, 179, 163, 153/152 (100%)/151, 135, 127	Protocatechuic acid arabinoyl/xyloyl glucoside/galactoside, *tent*.	+	+	+
13	4.75	327	705	513 (100%), 489, 339	Caffeoyl quinic acid dehydrodimer [41]	+	+	+
14	5.25	324 295sh	353	191 (100%)	Chlorogenic acid (standard)	+	-	-
15	5.51	323 289sh	705	595, 513 (100%), 485, 339	Caffeoyl quinic acid dehydrodimer [41]	-	-	+
16	5.64	325	179	143, 135 (100%), 71	Caffeic acid (standard)	+	+	-
17	6.26	256 293	457	371, 337, 283, 163 (100%)	n.i. hydroxycinnamic acid derivative	+	+	+
18	6.38	-	353	191 (100%)	Chlorogenic acid, isomer [42]	+	-	-
19	7.33	-	337	191 (100%), 173, 163	5-O-p-coumaroyl-quinic acid [42,43]	+	-	+
20	7.57	324	627	595, 447, 429 (100%), 183	n.i. phenolic acid derivative	+	+	+
21	8.57	258 355	595	343, 301 (100%), 271, 255, 179	Quercetin arabinosyl/xylosyl glucoside/galactoside (PubChem CID: 14054266 **)	+	+	-
22	8.83	258 357	463	301 (100%)	Hyperoside (standard)	+	+	-
23	8.95	260 355	609	301 (100%)	Rutin (standard)	+	+	-
24	8.97	258 356	463	301 (100%)	Isoquercitrin (standard)	+	-	-
25	9.11	265 342	579	447 (100%), 301	Quercetin arabnosyl/xylosyl rhamnoside, *tent*. [44]	-	-	+
26	9.37	255 364	433	301 (100%), 271, 179, 151	Quercetin arabinoside or xyloside [40]	+	+	+
27	9.50	265 348	579	447 (100%), 433, 301	Quercetin arabnoside/xyloside rhamnoside [44]	-	-	+
28	9.85	259 351	895	447 (100%), 301, 271	Quercetin rhamnoside derivative, *tent*.	+	+	+
29	10.00	258 351	447	301 (100%)	Quercetin rhamnoside (MB: PR100993 *)	+	+	+
30	10.81	266 344	431	285 (100%), 255, 227	Kaempferol rhamnoside (MB: PR100970 *)	+	+	+
31	11.05	257 371	301	273, 179 (100%), 151, 107	Quercetin (standard)	+	+	+
32	12.15	269 367	285	285 (100%), 225, 213, 199, 151	Kaaempferol (standard)	+	+	-
33	12.22	251 370	315	300 (100%)/301	Isorhamnetin (standard)	+	+	-
34	12.61	271	361	343 (100%), 317, 299	n.i.	-	-	+
35	12.99	-	361	343, 317 (100%), 299, 273	n.i.	+	-	+
36	13.25	272	351	336, 289 (100%), 271, 253, 241, 173	n.i.	+	+	+
37	15.10	-	265	97 (100%)	n.i.	+	+	+
38	15.43	279	309	263, 123, 97 (100%)	n.i.	+	+	+

* MB—Mass Bank at https://pubchem.ncbi.nlm.nih.gov, accessed 18 March 2024; ** PubChem CID—https://pubchem.ncbi.nlm.nih.gov, accessed 14 March 2024; n.i.—not identified; *tent*.—tentatively; sh—shoulder.

**Table 3:** Minimal inhibitory concentration (mg/mL) of *S. virgaurea* essential oils obtained by hydrodistillation (EGEO1), microwave-assisted hydrodistillation (EGEO2) and solvent-free microwave-assisted extraction (EGEO3).

Microorganism	*Klebsiella* *pneumoniae* ATCC 700603	*Proteus vulgaris* ATCC8427	*Escherichia coli* ATCC 25922	*Pseudomonas aerigunosa* ATCC 27853	*Staphylococcus aureus* ATCC 25923	*Bacillus subtilis* ATCC 6633	*Bacillus cereus* ATCC 11778	*Listeria* *monocytogenes* ATCC 15313	*Candida albicans* ATCC 2091
EGEO1	24.17 ± 1.44 a	24.17 ± 1.44 a	0.76 ± 0.05 a	25 ± 0.00 a	6.04 ± 0.36 b	6.04 ± 0.36 c	0.75 ± 0.05 a	1.51 ± 0.09 a	0.76 ± 0.04 a
EGEO2	23.33 ± 1.44 a	23.33 ± 1.44 a	3.02 ± 0.18 b	24.17 ± 1.44 a	3.02 ± 0.19 a	1.25 ± 0.41 a	0.99 ± 0.36 a	3.02 ± 0.18 b	0.76 ± 0.04 a
EGEO3	24.17 ± 1.44 a	24.17 ± 1.44 a	3.02 ± 0.18 b	25 ± 0.00 a	6.04 ± 0.36 b	3.02 ± 0.18 b	0.51 ± 0.24 a	3.02 ± 0.18 b	1.51 ± 0.09 b

a,b,c—different letters indicate statistically significant differences of the values in the same column (*p* < 0.05).

**Table 4:** Minimal inhibitory concentration (mg/mL) of *S. virgaurea* hydrolates obtained in the hydrodistillation (EGH1), microwave-assisted hydrodistillation (EGH2) and solvent-free microwave-assisted process (EGH3).

Microorganism	*Klebsiella* *pneumoniae* ATCC 700603	*Proteus vulgaris* ATCC8427	*Escherichia coli* ATCC 25922	*Pseudomonas aeruginosa* ATCC 27853	*Staphylococcus aureus* ATCC 25923	*Bacillus subtilis* ATCC 6633	*Bacillus cereus* ATCC 11778	*Listeria* *monocytogenes* ATCC 15313	*Candida albicans* ATCC 2091
EGH1	48.33 ± 2.89 b	48.33 ± 2.89 b	5.95 ± 0.33 a	46.67 ± 2.88 b	12.08 ± 0.72 b	24.17 ± 1.44 b	12.08 ± 0.72 a	6.04 ± 0.36 a	12.08 ± 0.72 a
EGH2	24.17 ± 1.44 a	24.17 ± 1.44 a	12.5 ± 0.00 b	24.17 ± 1.44 a	6.04 ± 0.36 a	12.08 ± 0.72 a	12.50 ± 0.00 a	24.17 ± 1.44 c	12.08 ± 0.73 a
EGH3	24.17 ± 1.44 a	45.00 ± 5.00 b	12.12 ± 0.66 b	48.33 ± 2.88 b	12.08 ± 0.72 b	24.17 ± 1.44 b	12.08 ± 0.72 a	12.08 ± 0.72 b	12.08 ± 0.74 a

a,b,c—different letters indicate statistically significant differences of the values in the same column (*p* < 0.05).

**Table 5:** Antioxidant activity of *S. virgaurea* essential oil obtained by hydrodistillation (EGEO1), microwave-assisted hydrodistillation (EGEO2) and solvent-free microwave-assisted extraction (EGEO3).

Essential Oil	DPPH Neutralization (%)
EGEO1	58.48 ± 0.05 a
EGEO2	62.23 ± 0.09 b
EGEO3	76.11 ± 0.11 c

a,b,c—different letters indicate statistically significant differences of the values in the same column (*p* < 0.05).

**Table 6:** Antioxidant activity of *S. virgaurea* L. hydrolates obtained in the hydrodistillation (EGH1), microwave-assisted hydrodistillation (EGH2) and solvent-free microwave-assisted process (EGH3).

Hydrolates	DPPH Neutralization (%)	TPC (mg GAE/g)
EGH1	35.71 ± 0.10 a	4.56 ± 1.14 a
EGH2	45.75 ± 0.05 b	5.26 ± 0.90 b
EGH3	50.32 ± 0.08 c	7.83 ± 1.09 c

a,b,c—different letters indicate statistically significant differences of the values in the same column (*p* < 0.05).

## Data Availability

The original contributions presented in the study are included in the article, further inquiries can be directed to the corresponding author.
